# Effect of intravenous vs. inhaled penehyclidine on respiratory mechanics in patients during one-lung ventilation for thoracoscopic surgery: a prospective, double-blind, randomised controlled trial

**DOI:** 10.1186/s12890-023-02653-8

**Published:** 2023-09-19

**Authors:** Ming-zi An, Cheng-yun Xu, Yue-ru Hou, Zhen-ping Li, Te-sheng Gao, Qing-he Zhou

**Affiliations:** 1 Anesthesia Medicine, Jiaxing University Master Degree Cultivation Base, Zhejiang Chinese Medical University, Hangzhou, Zhejiang Province China; 2 Department of anaesthesiology, Jiaxing Chinese Medical Hospital, No. 1501, Zhongshan East Road, Jiaxing, Zhejiang Province China; 3 Department of anaesthesiology and pain medicine, affiliated hospital of Jiaxing University, No.1882, South Central Road, Jiaxing, Zhejiang Province China

**Keywords:** Driving pressure, Mechanical power, Postoperative pulmonary complications, Penehyclidine

## Abstract

**Background:**

Minimising postoperative pulmonary complications (PPCs) after thoracic surgery is of utmost importance. A major factor contributing to PPCs is the driving pressure, which is determined by the ratio of tidal volume to lung compliance. Inhalation and intravenous administration of penehyclidine can improve lung compliance during intraoperative mechanical ventilation. Therefore, our study aimed to compare the efficacy of inhaled vs. intravenous penehyclidine during one-lung ventilation (OLV) in mitigating driving pressure and mechanical power among patients undergoing thoracic surgery.

**Methods:**

A double-blind, prospective, randomised study involving 176 patients scheduled for elective thoracic surgery was conducted. These patients were randomly divided into two groups, namely the penehyclidine inhalation group and the intravenous group before their surgery. Driving pressure was assessed at T_1_ (5 min after OLV), T_2_ (15 min after OLV), T_3_ (30 min after OLV), and T_4_ (45 min after OLV) in both groups. The primary outcome of this study was the composite measure of driving pressure during OLV. The area under the curve (AUC) of driving pressure from T_1_ to T_4_ was computed. Additionally, the secondary outcomes included mechanical power, lung compliance and the incidence of PPCs.

**Results:**

All 167 participants, 83 from the intravenous group and 84 from the inhalation group, completed the trial. The AUC of driving pressure for the intravenous group was 39.50 ± 9.42, while the inhalation group showed a value of 41.50 ± 8.03 (*P* = 0.138). The incidence of PPCs within 7 days after surgery was 27.7% in the intravenous group and 23.8% in the inhalation group (*P* = 0.564). No significant differences were observed in any of the other secondary outcomes between the two groups (all *P* > 0.05).

**Conclusions:**

Our study found that among patients undergoing thoracoscopic surgery, no significant differences were observed in the driving pressure and mechanical power during OLV between those who received an intravenous injection of penehyclidine and those who inhaled it. Moreover, no significant difference was observed in the incidence of PPCs between the two groups.

## Introduction

Postoperative pulmonary complications (PPCs) are commonly observed during the first postoperative week and have been associated with a prolonged hospital stay [1], increased hospital costs [2], and increased patient mortality rates [3]. In thoracic surgery, particularly during one-lung ventilation (OLV), the incidence of PPCs is comparable to that observed in abdominal surgery [4]. Certain patient factors, such as pre-existing lung disease, lung resection, extensive surgical trauma, reduced lung function, or the need for OLV, significantly increase the risk of developing complications [5–8]. Respiratory PPCs include pleural effusion, pneumothorax, pneumonia, respiratory failure, bronchospasm, and pulmonary atelectasis [9].

Strategies aimed at decreasing the incidence of PPCs include preoperative functional exercise [10], intraoperative protective lung ventilation [11], pulmonary resuscitation [12], and the use of respiratory medications throughout the perioperative period. Anticholinergic agents during the perioperative period have shown efficacy in reducing the incidence of PPCs. Inhaled tiotropium bromide has been found to potentially lower closed volume and glandular secretion, leading to improved tolerance of intraoperative mechanical ventilation [13–15]. Penehyclidine, a novel anticholinergic drug, which selectively antagonises M1 and M3 receptors, has demonstrated benefits in reducing airway hyperresponsiveness, inhibiting inflammatory responses, and enhancing lung compliance [16]. Previous studies have demonstrated that inhaled and intravenous penehyclidine can effectively reduce PPCs [17]. However, a direct comparison between these two routes of administration has not been undertaken to establish their equivalence.

Airway driving pressure and mechanical power are two widely used parameters in pulmonary protective ventilation. Airway driving pressure represents the pressure generated by mechanically ventilated patients during inhalation and is expressed as the ratio of tidal volume to lung compliance. It can also be calculated at the bedside by subtracting positive end-expiratory pressure (PEEP) from platform pressure [18]. A recent meta-analysis has demonstrated that driving pressure is independently associated with PPCs [19]. Additionally, a prospective study has demonstrated that an individualised ventilation strategy based on titrating driving pressure can reduce PPCs by approximately 7% during OLV compared with conventional protective ventilation [20]. Mechanical power is a new concept in mechanical ventilation, quantifying the energy delivered to the respiratory system and lung during mechanical ventilation, measured in Joule per minute (J/min) [21–25]. Several retrospective studies have shown that mechanical power is also a significant risk factor for PPCs [26, 27]. However, the lack of randomised controlled studies in this area is attributed to the complexity involved in calculating mechanical power, which requires considering multiple factors.

This clinical trial evaluated the specific roles of driving pressure and mechanical power as mediators in reducing PPCs. A randomised, prospective, and double-blind study was conducted to determine whether inhaled penehyclidine could effectively decrease the incidence of PPCs by reducing individualised drive pressure and mechanical power when compared with intravenous penehyclidine.

## Materials and methods

This prospective trial was conducted at affiliated hospital of Jiaxing University, China, from September 2022 to April 2023. The Institutional Review Board approved this study (2022-LY-164), and it was registered with the Chinese Clinical Trial Registry (www.chictr.org.cn, ChiCTR2200063427; 06/09/2022). Written informed consent was obtained from all participants before their inclusion in the study.

### Study population

The study included patients aged over 50 years who were undergoing thoracic surgery with OLV lasting expected to more than 45 min. Participants were required to have an American Society of Anaesthesiologists physical status of 1–3 and a postoperative hospital stay of at least 3 days to be eligible for participation. Patients were excluded from the study if they met any of the following criteria: (1) inability to cooperate during inhalation therapy, (2) moderate-to-severe symptomatic prostatic hypertrophy or narrow-angle glaucoma, (3) history of a previous myocardial infarction, severe heart dysfunction (New York Heart Association classification > 3), or tachyarrhythmia within the past 3 months, (4) presence of severe respiratory tract infections with low and thick sputum, (5) severe renal insufficiency requiring renal replacement therapy, (6) severe liver dysfunction (Child–Pugh class C), (7) recent use of anticholinergic drugs on the day before surgery, (8) prohibition from using PEEP, and (9) refused to participate in the trial. Additionally, patients taking other intraoperative anticholinergic drugs, those whose surgeries were cancelled, and patients who experienced serious allergies during surgery were considered as dropout criteria.

### Blinding and randomisation

The anaesthesia was administered by an anaesthesiologist who was blinded to the patient grouping, and the postoperative assessment was conducted by a researcher who was also blinded to the patient grouping. All patients received a combination of inhalation and intravenous therapy. In both groups, the study drug, 0.5 mg penehyclidine, was diluted and mixed with 5 mL of normal saline. In group A, patients inhaled penehyclidine with a fraction of inspired oxygen (FiO_2_) of 5–8 mL/kg within 15–20 min before surgery, and 5 mL of normal saline was injected before anaesthesia induction. On the other hand, patients in group B inhaled 5 mL of normal saline with a FiO_2_ of 5–8 mL/kg approximately 15–20 min before surgery, and 5 mL of penehyclidine was injected before the anaesthesia induction.

An independent investigator generated a computer-based randomisation list. The participants were randomly assigned to the inhalation or intravenous group in a 1:1 ratio using a computer-generated random sequence. The randomisation process involved sealed, sequentially numbered, and opaque envelopes that were kept in the operating room.

### Procedures

The patient’s information was thoroughly reviewed by the surgeon and nurse before the start of the procedure. Intraoperative monitoring equipment, such as electrocardiogram, oxygen saturation, invasive arterial blood pressure, end-expiratory carbon dioxide, airway pressure, and entropy index, was also meticulously checked to ensure proper connections and functionality. The general anaesthesia approach typically involved a combination of inhalation and intravenous medications, comprising a bolus of propofol (1.5–2.5 mg/kg), rocuronium (0.6–0.8 mg/kg) and sufentanil (0.2–0.5 µg/kg). During the maintenance phase of anaesthesia, sevoflurane, remifentanil, and propofol were used. An appropriate double-lumen tracheal tube was selected based on the patient’s sex (37 for males and 35 for females). The positioning of the double-lumen endotracheal tube and bronchi was determined using fibreoptic bronchoscopy. Additional medications, such as rocuronium, were administered as required during the surgery. The target entropy index was maintained between 40 and 60 during the maintenance phase of general anaesthesia. Anaesthesiologists were given discretion in the use of analgesic pumps or peripheral nerve blocks when possible. Lactated Ringer’s solution was administered as the maintenance fluid at a rate of 3–5 mL/kg/h. Intraoperative vasoactive drugs were administered based on the mean arterial pressure, and routine postoperative antiemetic medications, such as glasnost, were administered.

In this study, a standardised ventilation strategy was implemented for each patient. Tidal volume and respiratory rate were set at 6 mL/kg of predicted body weight and 13 breaths per minute, respectively, during OLV. Volume-controlled ventilation was employed with a 30% inspiratory pause and a 1:2 inspiratory to expiratory ratio. The inspired oxygen level was maintained at ≥ 60% and mixed with air. Predicted body weight (PBW) was calculated based on the patient’s sex, with PBW for men calculated as 50 + 0.91 × (height [cm] − 152.4) and PBW for women calculated as 45.5 + 0.91 × (height [cm] − 152.4). Oxygen concentration was increased as required to ensure adequate oxygenation during OLV and maintain a saturation level of at least 95%. After 5 min of OLV, the lowest driving pressure was determined, and PEEP was gradually increased from 0 to 10 cmH_2_O. Each level of PEEP was consistently maintained for eight respiratory cycles, and the driving pressure was recorded during the final cycle of each PEEP level for better accuracy. The PEEP level that resulted in the lowest driving pressure was selected to maintain a consistent PEEP level during OLV. Prior to the incision, a lateral position trial was conducted to determine the PEEP level associated with the lowest driving pressure.

### Data collection and outcome assessment

For data collection, a standardised form was used, sourced from the clinical charts to obtain baseline characteristics. Baseline data included demographic and morphometric characteristics, preoperative comorbidities, and smoking and alcohol history, along with pulmonary function test results. These pulmonary function test results were categorised based on clinical diagnosis and their reported values: 1) mild (forced expiratory volume in 1 s [FEV_1_] ≥ 80% of the predicted value, 2) moderate (50% of the predicted value ≤ FEV_1_ < 80% of the predicted value), 3) severe (30% of the predicted value ≤ FEV_1_ < 50% of the predicted value), 4) very severe (FEV_1_ < 30% of the predicted value).

Intraoperative data included the types and doses of anaesthetics/medications, anaesthesia duration, fluid balance, mechanical ventilation settings, OLV duration, use of vasoactive drugs (such as ephedrine, phenylephrine, and metaraminol), the surgery type and duration, and the surgical site.

The primary outcome of this study was the composite measure of driving pressure, which was assessed at several specific time intervals: T_1_ (5 min after OLV), T_2_ (15 min after OLV), T_3_ (30 min after OLV), and T_4_ (45 min after OLV). The driving pressure was calculated using the following equation: driving pressure (cmH_2_O) = plateau pressure − PEEP. The area under the curve (AUC) of driving pressure from T_1_ to T_4_ for the participant was calculated. The composite secondary outcome was the occurrence of major PPCs within 7 days after surgery, including respiratory infection, respiratory failure, pleural effusion, atelectasis, pneumothorax, bronchospasm, and hypoxaemia [4]. These complications were classified as grade II or above based on the Clavien–Dindo classification [28]. In addition to PPCs, several other secondary outcomes related to respiratory mechanics, such as mechanical power, the AUC of the mechanical power from T_1_ to T_4_, lung compliance, and adverse drug reactions associated with penehyclidine were evaluated. Adverse drug reactions associated with penehyclidine included dry mouth, skin rash, pupil dilation, dizziness, urinary retention, and elevated body temperature. These measures were assessed concurrently, providing a comprehensive perspective of respiratory function. The formula used to calculate mechanical power (in J/min) was 0.098 × tidal volume × respiratory rate × (peak pressure − [0.5 × driving pressure]) [24].

### Sample size estimation

During the preliminary study, the driving pressure of 60 patients across T_1_-T_4_ intervals was evaluated to increase the accuracy of our sample size calculation. The AUC of driving pressure was determined during the procedure for both groups, resulting in values of 41.40 ± 8.14 for the inhalation group and 37.48 ± 9.22 for the intravenous group. A total of 158 patients were recruited for this study to achieve a two-sided alpha level of 5% and a statistical power of 80%. Considering a dropout rate of 10%, the target enrolment was established at 176 patients, with each group comprising 88 participants.

### Statistical analysis

Categorical variables are presented as numbers and percentages. Continuous variables are presented as the mean ± standard deviation or median (interquartile). The chi-square test or Fisher’s exact test was used to compare categorical variables and Student’s t-test or Mann–Whitney U test was used for continuous variables, based on the normality of the data. Generalised estimating equations were used for repeated measures. Statistical analyses were performed using SPSS version 25 and GraphPad Prism 9, with a significance level set at 0.05 (two-tailed).

## Results

**Fig. 1:**
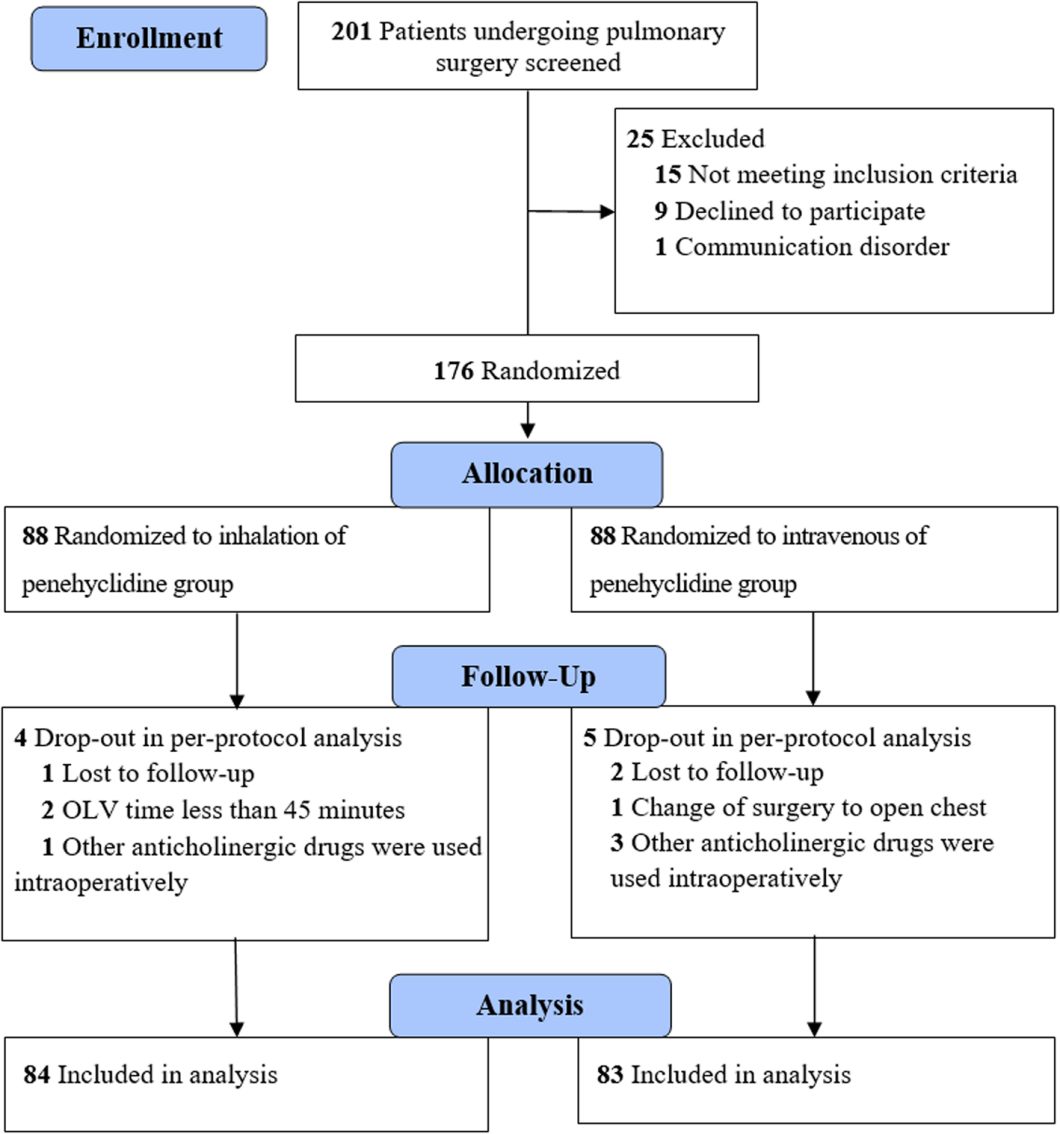
Flow chart for patients undergoing thoracoscopic respiratory mechanical studies

From September 2022 to April 2023, 201 patients were initially assessed for eligibility, of which 176 patients were randomised. Nine patients were excluded after randomisation, resulting in 167 patients for per-protocol analysis. The final analysis encompassed 83 patients in the intravenous group and 84 patients in the inhalation group (Fig. 1). The baseline characteristics of the two groups were well balanced at randomisation (Table 1). There were no statistically significant differences between intraoperative and postoperative characteristics (Table 2).

**Table 1:** Demographic and baseline data

	Intravenous group (*n* = 83)	Inhalation group (*n* = 84)	*P-*value
Age, years	66.0(59.0–70.0)	61.0(57.0–67.0)	0.018
Male/Female, n	33/50	30/54	0.590
Weight, kg	58.5(53.0–67.0)	62.0(55.0-68.2)	0.175
Height, cm	160.0(155.0-165.0)	160.0(156.0-165.0)	0.410
BMI, kg/m^2^	22.9(20.9–26.1)	24.0(22.2–25.8)	0.116
Chronic disease, n (%)			
Respiratory diseases	6(7.2%)	2(2.4%)	0.269
Hypertension	45(54.2%)	46(54.8%)	0.944
Diabetes	14(16.9%)	11(13.1%)	0.495
Heart disease	5(6.0%)	4(4.8%)	0.985
Drinking, n (%)	2(2.4%)	4(4.8%)	0.689
Smoking, n (%)	9(10.8%)	6(7.1%)	0.403
ASA, classification, n (%)			0.368
I	1(1.2%)	0(0%)	
II	65(78.3%)	71(84.5%)	
III	17(20.5%)	13(15.5%)	
Preoperative pulmonary function			0.888
Normal, n (%)	59(71.1%)	57(67.9%)	
Mild, n (%)	20(24.1%)	23(27.4%)	
Moderate to severe, n (%)	4(4.8%)	4(4.8%)	
FVC, L	2.79(2.27–3.32)	2.71(2.22–3.43)	0.930
FEV_1_, %	98.6(88.8–107)	96.8(84.0-109.0)	0.734
FEV_1_/FVC, %	79.0(73.9–83.7)	79.6(72.0-84.3)	0.973
DLCO_2_, %	84.1(75.8–95.7)	84.8(77.0-92.6)	0.846

*ASA *American Society of Anaesthesiologists physical status, *BMI *Body mass index, *DLCO*_2 _ Diffusing capacity of carbon dioxide, *FEV*_1_ Forced expiratory volume in 1 s, *FVC* Forced vital capacity

**Table 2:** Intraoperative and postoperative characteristics

Intraoperative	Intravenous group (*n* = 83)	Inhalation group (*n* = 84)	*P*-value
Medication during anaesthesia			
Propofol dosage, mg	200(200–370)	200(200–359)	0.921
Rocuronium dosage, mg	52.0(50.0–60.0)	56.0(50.0–60.0)	0.381
Sufentanil dosage, µg	30.0(30.0–30.0)	30.0(30.0–30.0)	0.670
Remifentanil dosage, µg	1000(1000–1750)	1000(1000–1200)	0.584
Sevoflurane dosage, mL	30.6(21.6–41.1)	27.0(20.4–35.2)	0.134
Vasoactive drugs, n (%)	37(44.6%)	34(40.5%)	0.592
Surgical procedure, n (%)			0.283
Wedge resection	27(32.5%)	19(22.6%)	
Segmentectomy	37(44.6%)	39(46.4%)	
Lobectomy	19(22.9%)	26(31.0%)	
Surgical site, n			
Left/Right	41/42	36/48	0.397
Duration of anaesthesia, min	105.0(85.0-135.0)	100.0(85.0-120.0)	0.393
Duration of surgery, min	85.0(67.0-120.0)	85.0(70.0-105.0)	0.558
Duration of OLV, min	72.0(60.0–95.0)	77.0(58.5–91.0)	0.794
Time to extubation, min	20.0(15.0–30.0)	20.0(10.0–25.0)	0.101
Total fluid input, mL	1000(1000–1500)	1000(1000–1000)	0.129
Tidal volume during OLV, mL/kg	6.12(5.64–6.74)	5.92(5.47–6.38)	0.121
PEEP during OLV, cmH_2_O	4(3–5)	4(3–5)	0.481
Intraoperative mini-MAP, mmHg	66.0(61.3–71.0)	66.7(60.9–69.2)	0.655
Time of chest drain removal, days	3(3–4)	3(3–4)	0.815

*MAP *Mean arterial pressure, *OLV *One-lung ventilation, *PEEP *Positive end-expiratory pressure

### Driving pressure

**Fig. 2:**
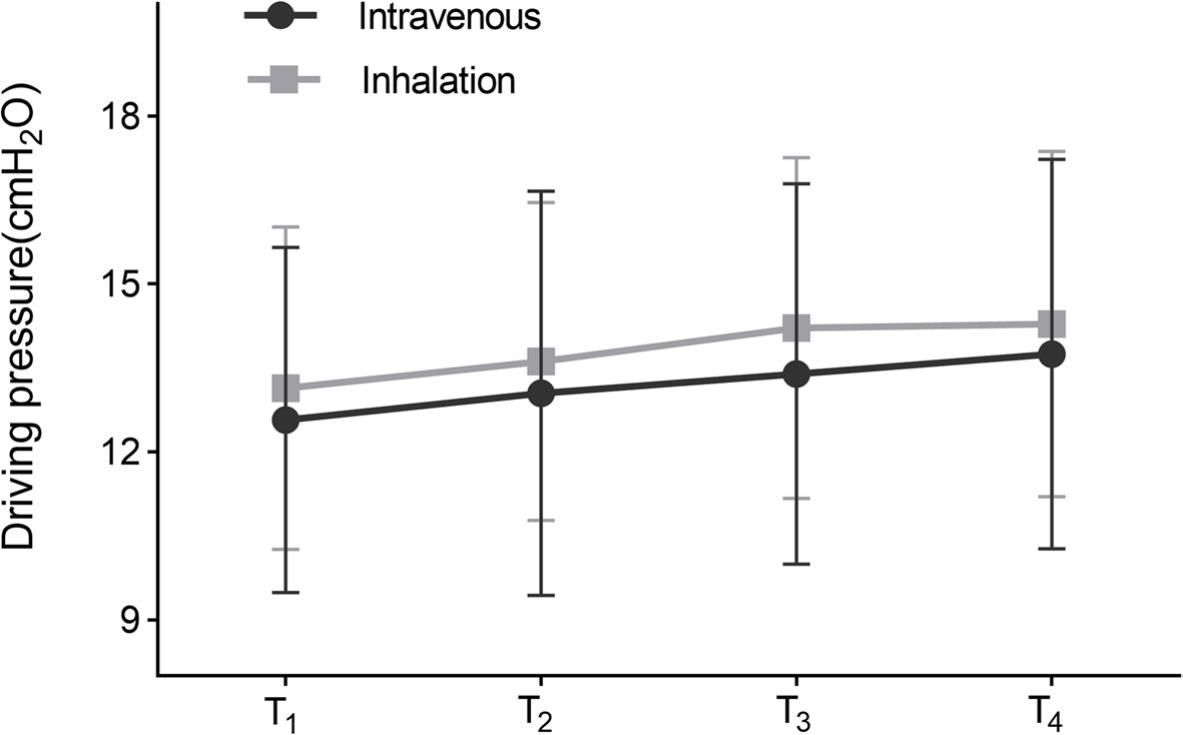
Driving pressure. According to generalised estimating equations, no statistically significant difference was observed in the driving pressure between the two groups (*P* = 0.144); Statistically significant differences in driving pressure were observed at T_1_, T_2_, T_3_, and T_4_ for the intravenous and inhalation groups (intragroup comparison) (both *P* < 0.001). T_1_, 5 min after OLV; T_2_, 15 min after OLV; T_3_, 30 min after OLV; T_4_, 45 min after OLV

The AUC for driving pressure did not reveal any significant differences between the inhalation and intravenous groups (41.50 ± 8.03 vs. 39.50 ± 9.42, *P* = 0.138) (Table 3). Similarly, according to generalised estimating equations, no significant difference in driving pressure from T_1_ to T_4_ was detected between the two groups (*P* = 0.144). At each time point, there was no significant difference in driving pressure within the inhalation group compared with the intravenous group. In terms of intragroup analysis, the intravenous and inhalation groups exhibited statistically significant differences in driving pressure at T_1_, T_2_, T_3_, and T_4_ (both *P* < 0.001) (Fig. 2). There was no interaction effect was observed between the group and time (*P* = 0.769).

**Table 3:** Driving pressure and mechanical power during OLV and PPCs

Outcome	Intravenous group (*n* = 83)	Inhalation group (*n* = 84)	*P*-value
AUC of the driving pressure	39.50 ± 9.42	41.50 ± 8.03	0.138
AUC of the mechanical power	24.20(19.7–27.1)	24.00(20.7–27.9)	0.556
Incidence of PPCs within 7 days, n (%)	23(27.7%)	20(23.8%)	0.564
Adverse drug reactions associated with penehyclidine, n (%)	3(3.6%)	1(1.2%)	0.604

*AUC *Area under the curve, *OLV *One-lung ventilation, *PPCs *Postoperative pulmonary complications

### Mechanical power

**Fig. 3:**
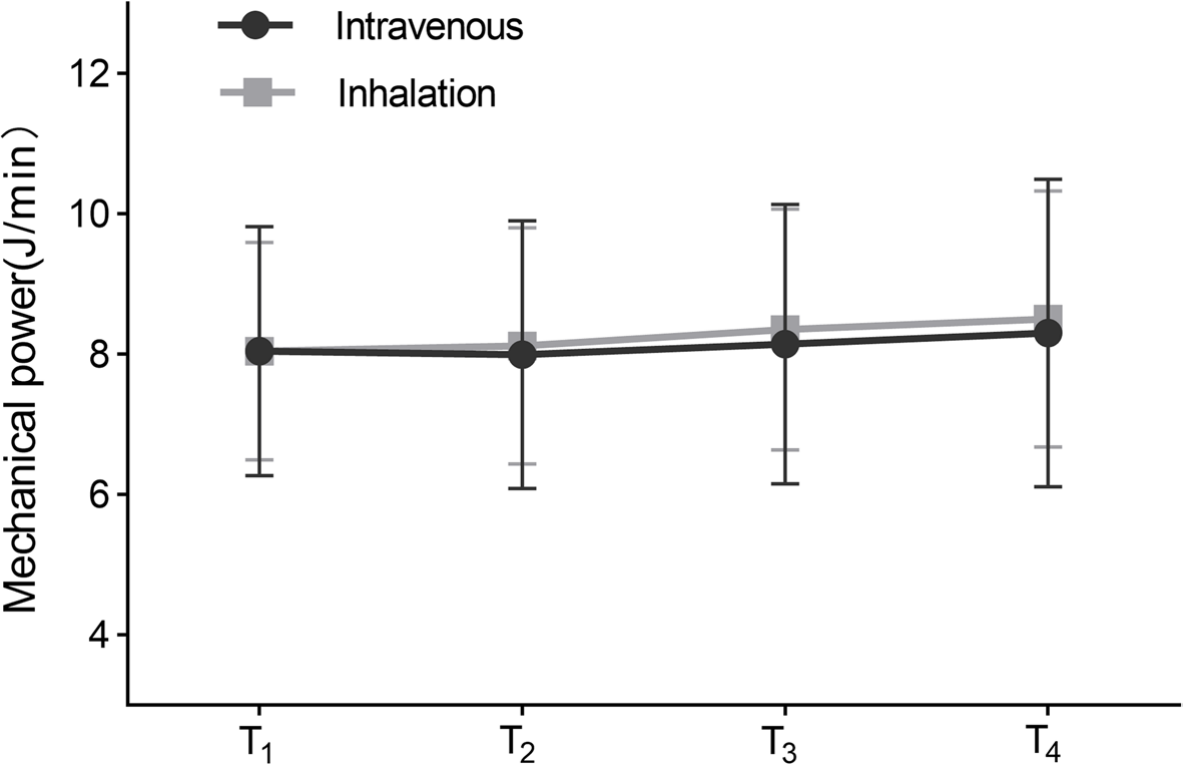
Mechanical power. According to generalised estimating equations, the mechanical power did not differ significantly in the intergroup comparison (*P* = 0.545); A significant difference was observed only in the intragroup comparison of the inhalation group in mechanical power at T_1_, T_2_, T_3_, and T_4_ (intravenous group, *P* = 0.061; inhalation group, *P* < 0.001). T_1_, 5 min after OLV; T_2_, 15 min after OLV; T_3_, 30 min after OLV; T_4_, 45 min after OLV

No significant differences were observed between the inhalation and intravenous groups in terms of the AUC of mechanical power (24.00 [20.7–27.9] vs. 24.20 [19.7–27.1]; *P* = 0.556) (Table 3). Similarly, according to generalised estimating equations, no significant difference in mechanical power from T_1_ to T_4_ was observed between the two groups during the intergroup comparison (*P* = 0.545). Furthermore, when comparing the inhalation and intravenous groups at each time point, the differences in mechanical power were not statistically significant. In terms of intragroup comparisons, the inhalation group exhibited a significant overall difference in mechanical power (*P* < 0.001) (Fig. 3). Similar to the findings for driving pressure, there was no detected interaction effect between the group and time concerning mechanical power (*P* = 0.597).

### Secondary outcomes

**Fig. 4:**
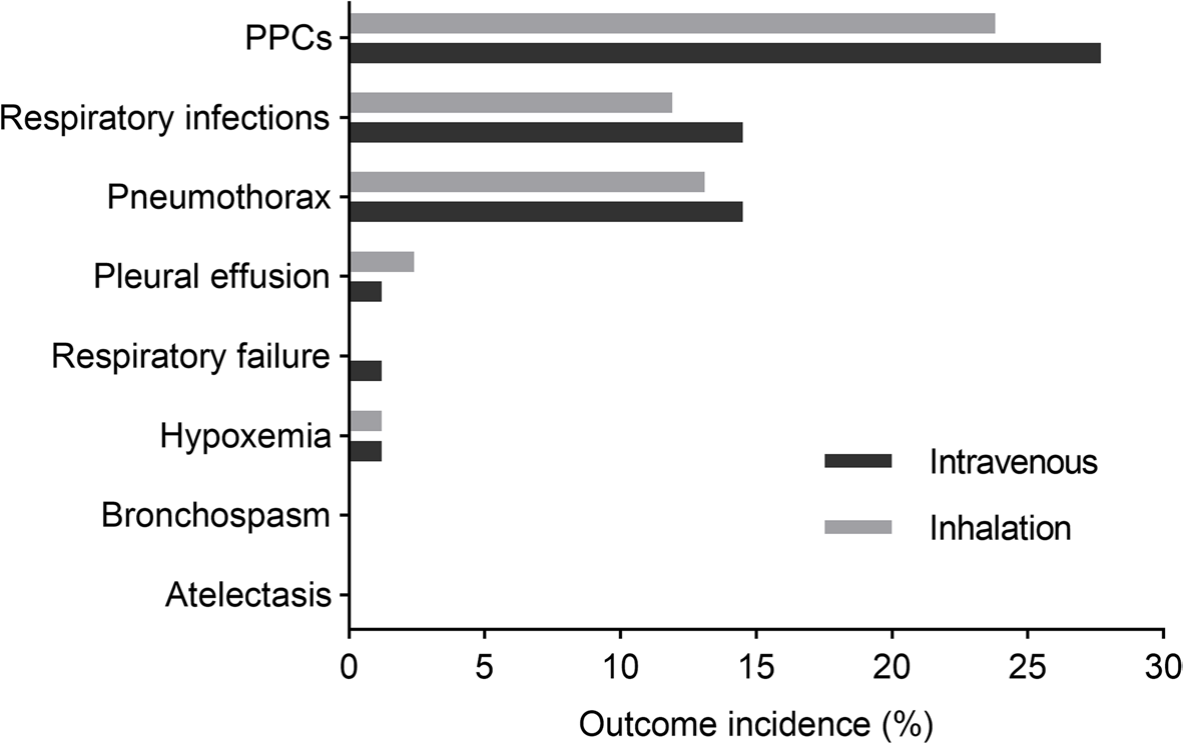
Incidence of postoperative pulmonary complications (PPCs) within 7 days. The incidence of PPCs was 23.8% among patients in the inhalation group compared with 27.7% in the intravenous group within 7 days postoperatively (*P* = 0.564)

Within 7 days postoperatively, PPCs, categorised according to the Clavien–Dindo classification were observed in 20 of the 84 patients (23.8%) in the inhalation group and 23 of the 83 (27.7%) patients in the intravenous group (*P* = 0.564) (Table 3; Fig. 4). No significant differences were observed in the incidence of respiratory infections and pneumonia between the two groups (12 of 84 [13.5%] vs. 10 of 83 [12.5%], *P* = 0.626). Respiratory failure and hypoxaemia were rare in both groups, with a singular instance of respiratory failure in the intravenous group. Hypoxaemia was observed in both groups, while bronchospasm or severe atelectasis were not reported in either group.

**Fig. 5:**
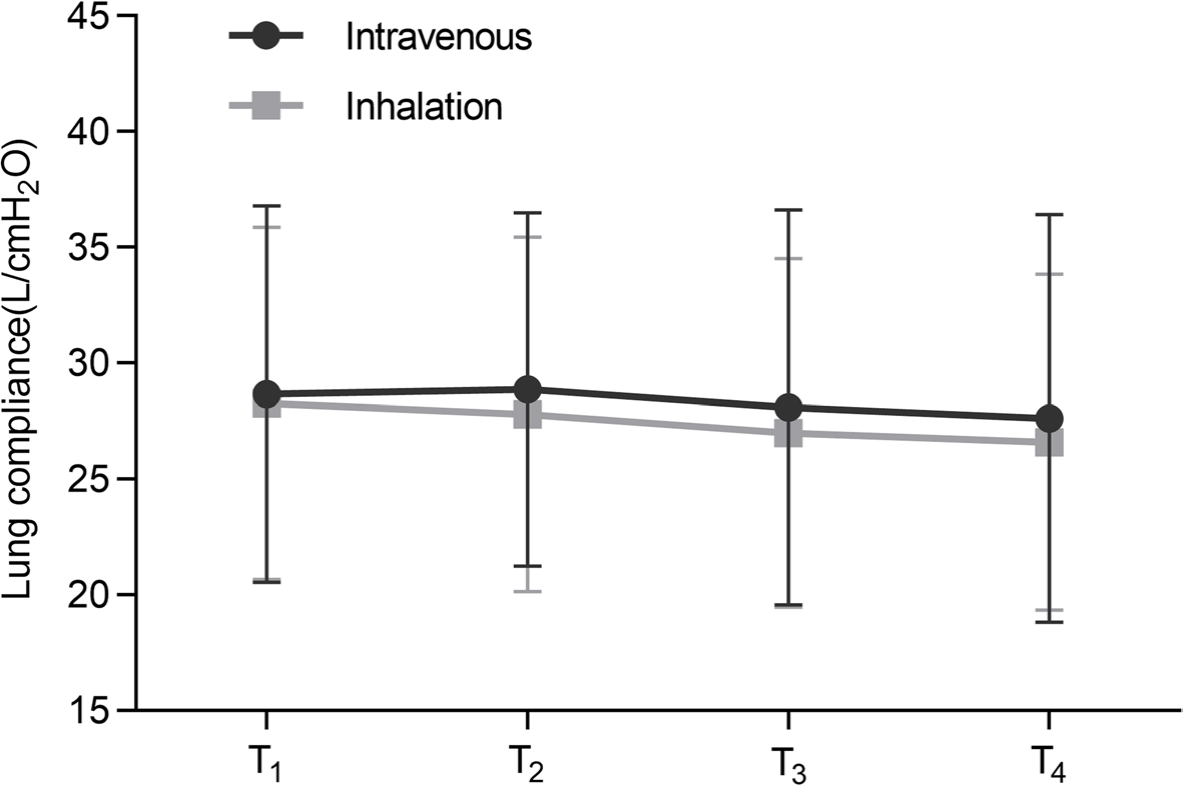
Lung compliance. According to generalised estimating equations, lung compliance between the two groups did not differ significantly in the intergroup comparison (*P* = 0.413). No significant difference was observed in the intragroup comparisons (intravenous group: *P* = 0.378; inhalation group: *P* = 0.095). T_1_, 5 min after OLV; T_2_, 15 min after OLV; T_3_, 30 min after OLV; T_4_, 45 min after OLV

According to generalised estimating equations, the comparison of lung compliance between the two groups was not significantly different in the intergroup comparison (*P* = 0.413). Similarly, no statistically significant distinctions were found between the inhalation and intravenous groups at each specific time point. In terms of the intragroup comparison, no significant differences were observed in the overall lung compliance (intravenous group: *P* = 0.378; inhalation group: *P* = 0.095) (Fig. 5). Furthermore, no interaction effect was observed between the group and time with regard to lung compliance (*P* = 0.766).

### Safety outcomes

No significant differences were observed in terms of the adverse reactions associated with penehyclidine, and no serious adverse outcomes were attributed to the study drug (Table 3).

## Discussion

In this randomised clinical trial involving adults undergoing thoracoscopic surgery, preoperative prophylactic inhalation of penehyclidine did not significantly reduce the driving pressure and mechanical power during OLV compared with intravenous administration. Moreover, there was no observed reduction in PPCs within the first 7 days postoperatively.

Preliminary studies have established an association between increased driving pressure and an augmented risk of complications and mortality [29, 30]. Our study addressed this concern by implementing an individualised strategy focused on the lowest driving pressure for protection [31]. Recent randomised trials have demonstrated the potential of driving pressure ventilation strategies to mitigate PPCs [20, 32]. However, a multicentre study revealed no statistically significant difference between a driving pressure-guided ventilation strategy and a protective PEEP ventilation strategy in thoracic surgery [33]. In our study, driving pressure was employed as a tool to explore the protective mechanism of penehyclidine. Our findings indicate that the method of administering penehyclidine was not significantly associated with driving pressure. However, notable overall differences in driving pressure were observed at the four time points between the inhalation and intravenous groups. With an increase in the duration of OLV, the driving pressure gradually increased, suggesting a possible correlation between the lowest driving pressure and the extent and duration of lung collapse.

Previous retrospective studies have drawn attention to a concerning connection between the excessive application of mechanical power during surgery and an increased risk of postoperative complications [26, 27]. Mechanical power refers to the energy necessary for ventilating the lungs and sustaining respiratory functionality. In clinical practice, there are two primary methods employed to mitigate mechanical power. The first entails reducing drive pressure or adjusting the respiratory rate, while the second involves employing low tidal volume ventilation [26]. In our study during OLV, a respiratory rate of 13 breaths per minute and a tidal volume of 6 mL/kg were established based on PBW. Our findings revealed no significant difference in driving pressure and mechanical power between the groups. This suggests that the lung protection attributed to penehyclidine is not significantly influenced by the method of administration. Nevertheless, in our study, the overall mechanical power of the inhalation group displayed a gradual increase throughout OLV.

Penehyclidine, a novel anticholinergic drug, specifically targets M1 and M3 receptors and achieves peak plasma concentration around 0.56 h after administration [16]. A prospective randomised controlled trial involving 864 participants demonstrated that inhaling penehyclidine could significantly decrease the occurrence of PPCs among high-risk individuals [17]. Additionally, routine intravenous penehyclidine during OLV was found to confer pulmonary protective benefits, particularly in elderly patients. Contrastingly, our study did not yield apparent advantages from inhaled penehyclidine in terms of reducing the incidence of PPCs. This might be attributed to the limited number of nebulised inhalations in our study, with only one administered before surgery. In contrast, other studies employed a regimen of seven inhalations before and after surgery. While there was a higher incidence of respiratory tract infections, pneumonia, and other complications, these occurrences did not hold clinical significance. Notably, though temporary dips in oxygen saturation were observed during surgery, they normalised after bilateral lung ventilation.

Nebulised drug inhalation delivers the drug directly to the airway, resulting in a higher local concentration and faster onset of action, with fewer systemic adverse effects. However, the absence of a significant difference between the inhaled and intravenous groups in our study might be attributed to the inhalation technique employed and the drug’s blood concentration. Further research should focus on determining the optimal dosage and inhalation method for nebulised drugs.

There are several limitations to this study. First, the absence of a placebo-controlled trial to demonstrate the effectiveness of penehyclidine on driving pressure and mechanical power is notable. This limitation stems from the requirement of routine anticholinergic administration during thoracic surgery to manage secretions. Second, our inability to determine the blood concentration and peak time after penehyclidine inhalation posed challenges in aligning the duration of action with intravenous administration. Third, this study concentrated on determining the driving pressure and mechanical power during OLV, however, it did not factor in the respiratory mechanics during two-lung ventilation, which could influence the reliability of the research results. Fourth, wedge resection has been found to have a low rate of PPCs in thoracic surgery [34]. The intravenous group had more wedge resections than the inhalation group in the current study, although the total PPCs between the two groups were not significantly different, which may lead to some degree of bias.

## Conclusions

The study revealed no significant differences in terms of driving pressure and mechanical power during OLV between intravenous injection and those receiving inhaled penehyclidine in the context of thoracoscopic surgery. Furthermore, there existed no difference in the incidence of PPCs between the two methods. Future research might explore the relationship between penehyclidine, driving pressure, and mechanical power in individuals with pre-existing pulmonary conditions or those at high risk of developing PPCs.

## Data Availability

All the data and material generated during the current study are available from the corresponding author upon reasonable request (zqh10980@zjxu.edu.cn).
